# A feasibility study for a randomised controlled trial of the Positive Reappraisal Coping Intervention, a novel supportive technique for recurrent miscarriage

**DOI:** 10.1136/bmjopen-2014-007322

**Published:** 2015-04-15

**Authors:** Sarah Bailey, Chris Bailey, Jacky Boivin, Ying Cheong, Isabel Reading, Nick Macklon

**Affiliations:** 1 Faculty of Health Sciences, University of Southampton, Southampton, UK; 2 School of Psychology, Cardiff University, Cardiff, UK; 3 Department of Obstetrics and Gynaecology, Academic Unit of Human Development and Health, University of Southampton, Southampton, UK; 4 Faculty of Medicine, University of Southampton, Southampton, UK

**Keywords:** GYNAECOLOGY

## Abstract

**Introduction:**

Recurrent miscarriage (RM) is diagnosed when a woman has had three or more miscarriages. Increased levels of distress and anxiety are common during the waiting period of any subsequent pregnancies, posing a significant threat to psychological well-being. However, only limited support and therapy are available for these women, and many are left to cope alone. The Positive Reappraisal Coping Intervention (PRCI) is a novel self-administered supportive technique which has been shown to be effective in patients awaiting the outcome of in vitro fertilisation treatment. The primary objective of this study is to assess the feasibility and effectiveness of the PRCI in improving quality of life in the difficult waiting period which women with previous RM endure before an ongoing pregnancy can be confirmed.

**Methods and analysis:**

A randomised controlled trial (RCT) feasibility study will establish the viability of conducting a multicentre RCT to definitively test the effects of the PRCI on the psychological well-being of women who have experienced RM during the initial waiting period of a subsequent pregnancy. A second component consists of a qualitative process evaluation exploring the initial experience of pregnancy following repeated miscarriages. Participants (n=50) will be randomised into one of two groups. The PRCI intervention group will receive the PRCI card and weekly questionnaires to assess their psychological well-being during the waiting period of their new pregnancy. The non-intervention group will be asked to complete the same weekly questionnaires. The qualitative process analysis will employ semistructured interviews (n=20) to address relevant aspects of the study objectives.

**Ethics and dissemination:**

Ethics approval has been obtained from the National Research Ethics Service Committee South Central—Hampshire A. Participating centres have given National Health Service R&D approval. Study findings will be disseminated through peer reviewed journals, national and international conferences and lay user groups.

**Trial registration number:**

ISRCTN43571276. This study was registered with the ISRCTN 18/02/2014 following adoption onto the United Kingdom Clinical Research Network (UKCRN) portfolio. Recruitment of the first participant occurred 04/02/2014.

**Strengths and limitations of this study**

- This study will test a validated tool in a randomised controlled trial providing essential feasibility information to inform a definitive fully powered study.
- Addresses a novel area of research not previously investigated.
- This mixed-method study uses participant qualitative interviews to supplement feasibility data providing additional insight into how women experience the waiting period of a new pregnancy following recurrent miscarriage.
- Feasibility study not powered for a definitive study.

## Background

Miscarriage is the most common adverse outcome of pregnancy^1^ and recurrent miscarriage (RM), defined as the loss of three or more consecutive pregnancies,^2^ affects approximately 1% of women. RM is a distressing and traumatic condition, representing more than a loss of a pregnancy; it evokes feelings surrounding a lost baby, a lost future child and a lost motherhood.^3^ Feelings of grief and depression are common^4–6^ and previous studies have suggested that single and RM can cause the affected woman a ‘significant physical and psychological challenge’^7^ posing a considerable threat to psychological well-being.^8^ Despite these potential grieving responses, women who experience miscarriage tend not to receive the same level of psychosocial support afforded to people experiencing other types of bereavement.^1^

Studies investigating emotional morbidity in women in the time period following miscarriage^9–11^ have indicated that increased levels of anxiety and depression are often experienced by women with a history of reproductive loss during subsequent pregnancies.^4^ ^8^ However, research data pertaining to the psychological morbidity associated with the difficult period in a subsequent pregnancy as the woman waits to see whether the pregnancy will again end in miscarriage or not, is scarce. The experience of RM frequently results in a period of ‘marked stress reaction’^12^ when the woman becomes pregnant again, specifically during the ‘waiting period’ between a positive pregnancy test and confirmation that the pregnancy is ongoing. Indeed, some women will elect not to conceive at all rather than repeatedly face this period of troubling uncertainty.

### The medical waiting period

Waiting periods have been defined as the period of time in which patients are waiting for medical test results which could be potentially threatening to their well-being.^13^ The medical waiting period can be a psychologically stressful time^14^ and Osuna^15^ proposes that during the waiting period a psychological reaction can occur whereby anticipation of loss leads to anxiety and potential psychological distress. The medical waiting period has been studied among a range of patient groups including women waiting for genetic information,^16^ women waiting for a breast cancer diagnosis,^17^ patients waiting for gastrointestinal endoscopy investigations,^18^ and the waiting period during fertility treatment between embryo transfer (in vitro fertilisation, IVF) and pregnancy test.^19^ A further study by Boivin and Lancastle^13^ has shown that medical waiting periods have a distinct emotional signature.

### The waiting period of a new pregnancy

In the context of a pregnancy after RM, the waiting period refers to the first 12 weeks of a pregnancy when women wait for ultrasound confirmation that their pregnancy is ongoing. This can be a particularly stressful and anxious time as the worry for the affected woman and her partner often increases substantially. Any excitement brought about by a positive pregnancy test is often overshadowed by the fear and despair that they will suffer yet another miscarriage. Once the woman has reached 12 weeks of pregnancy and an ultrasound scan has confirmed this, the chance of progression to term is around 95% and this reassurance is likely to make her feel more confident in her continuing pregnancy, and will experience less anxiety and distress.

There is a small but growing body of research concerning the waiting period experienced by women who have suffered RM. Recent evidence from a qualitative focus group study,^3^ for example, provides insight into the coping styles and experiences of women who have suffered miscarriages, focusing on the initial waiting period (eg, weeks 1–12) of a subsequent pregnancy. Differences were found in the way women with one miscarriage and women with RMs appraised and assessed the waiting period, but the coping strategies utilised in both groups were similar. These were most commonly emotion focused, and included avoidance, the seeking of social support, distraction and the utilisation of positive appraisal.

### Positive reappraisal and the positive reappraisal coping intervention

Positive reappraisal coping is an active strategy^20^ ^21^ which aims to reduce the emotional morbidity of a stressful period by focusing on the positive aspects of a situation. Positive reappraisal means choosing to take account of good aspects alongside the negative aspects of a situation, appreciating that even the most challenging difficult situations will have some positive elements.^19^

Previous studies have identified that positive reappraisal has been successful at reducing stress levels for many patient populations including those suffering from breast cancer,^22^ myocardial infarction^23^ and brain injury,^24^ and in caregivers whose partners were terminally ill with AIDS.^25^ In recent years, a theoretically derived, short, coping, intervention based on the concept of positive reappraisal has been developed, initially for use during the time period in which women wait for the results of their pregnancy test following fertility treatment.^19^ This intervention, called the Positive Reappraisal Coping Intervention (PRCI) was designed to stimulate positive reappraisal coping during the waiting period between IVF and a pregnancy test and aimed to encourage the consideration of positive aspects of the situation and its meaning for the patient. The study^19^ concluded that the women favourably evaluated the PRCI and found it acceptable and beneficial in terms of supporting positive feelings during the IVF waiting period, and sustaining their efforts to cope.

One of the key advantages of the PRCI approach is that it is convenient for patients and is easily deliverable at negligible cost. It comprises of an explanatory leaflet describing positive reappraisal coping and its potential benefits as well as 10 statements printed on a laminated card that users read at least twice a day to stimulate the use of this form of coping. Since use of the PRCI does not require clinic or hospital attendance or the direct involvement of a carer or health professional, it is very low in cost. Lancastle and Boivin^19^ demonstrated that despite the fact that the women had minimal contact with clinical or research staff during the waiting period, the intervention was still positively evaluated.

For women who have experienced RM, the waiting period in the early stages of pregnancy shares many characteristics and stress factors with the waiting period after fertility treatment. Both waiting periods include outcomes which are unpredictable, outcomes over which the woman has no control, and a waiting period which lasts several weeks. Both groups of patients may experience an increase in anxiety and worry and acute uncertainty about the outcome of this waiting period. Significantly, the psychological strain experienced by both groups and their partners is compounded by concerns and doubts about their ability to carry a successful pregnancy, a future child and their ability to become parents. The two groups of women share closely analogous experiences, and similarities between the characteristics and stress factors experienced in the two types of waiting period suggest that the PRCI is potentially valuable as well as cost-effective intervention for women during the initial waiting period of a new pregnancy. This proposed randomised controlled trial (RCT) feasibility and acceptability study of the intervention is the next step in assessing the suitability of the PRCI as a coping intervention for women who have experienced RM.

## Plan of investigation

### Study design

A mixed-method, two-centre, randomised controlled feasibility study aims to establish the viability of conducting a multicentre RCT to definitively test the effects of the PRCI on the psychological well-being of women who have experienced RM during the initial waiting period (1–12 weeks) of a subsequent pregnancy. Part 1 of this study will address how acceptable the proposed methods of recruitment and randomisation are: whether it is possible to achieve acceptable recruitment and retention rates; whether the proposed data collection methods are appropriate; whether the study time-points are appropriate; and whether there is any preliminary effect of the PRCI.

Part 2 of the study consists of a qualitative process evaluation which aims to explore women’s subjective experiences of the study intervention and research methods (including study outcome measures), provide information to refine the study intervention (if required), and to strengthen in-depth understanding of the initial experience of pregnancy following repeated miscarriages.

### Intervention

The PRCI consists of an explanatory leaflet and a small card containing 10 positive reappraisal statements that aim to encourage users to redefine the waiting period more positively. The leaflet provides concise guidance on the use of the PRCI. Specifically, women are encouraged to read the PRCI at least twice a day, in the morning and in the evening, and any other time of day they feel the need. When reading the statements, women are instructed to consider and think about how each statement relates to them personally. They are advised that thinking about the positive aspects of a difficult situation does not mean pretending that ‘everything is wonderful’ when they do not think it is, or ignoring the negative aspects of a difficult situation, but taking account of the positive aspects alongside the more negative aspects of the situation. The positive aspects of the waiting period will differ depending on personal circumstances, and the leaflet gives examples (eg, focusing on the support and kindness friends and family have shown during their difficulties or how their relationship with their partner has strengthened because of the shared experience).

The statements on the card are general and do not refer to any one specific positive aspect, as individual users will have very different ideas about what is or is not positive. The women will be encouraged to put the small card in a purse or pocket so that they are able to remind themselves of the positive reappraisal techniques wherever and whenever they feel the need. The leaflet advises that the positive reappraisal technique can feel strange at first, but that the technique becomes easier the more it is practiced.

### Target population

The study population will consist of patients attending the Recurrent Miscarriage Clinic and the Early Pregnancy Unit at two regional hospitals in the south of England. Women who have experienced three or more miscarriages will be invited to participate, as this population is at greater risk of high levels of anxiety and distress, as described previously.

### Inclusion and exclusion criteria

Inclusion criteria will be: women with three or more miscarriages, women aged >18 years, and those who are willing and able to give written consent. Women will be excluded from this study if they do not speak English well enough to understand and complete study materials. This criterion is in place because the study materials (including PRCI) are not currently available in translation.

### Feasibility RCT study

#### Proposed sample size and sampling strategy

Since this is a feasibility study which will indicate treatment effect for the design of a definitive study, the sample size is not determined by a power calculation.

The study sample will consist of two groups: the intervention group will receive the PRCI in addition to the current recommended care pathway, and the control group will follow the current recommended care pathway. A maximum of 25 patients will be recruited to each of the treatment arms over a 12-month period. There will be a lag time from recruitment (when the women experience their most recent miscarriage) to randomisation (when the participants inform the study team that they have a positive pregnancy test). This period, however, is likely to be relatively short, as many women who experience RM become pregnant quickly and have a short ‘time-to-pregnancy’,^26^ despite difficulty carrying the pregnancy to term. Two of the aims of this study, however, are to investigate patient flow for an actual recruitment rate to this study and the time lag from recruitment to randomisation.

### Recruitment and randomisation

Potential participants will be given information about the study by their clinical care team when they attend the Recurrent Miscarriage Clinic or Early Pregnancy Unit and are invited to take part. If they agree, participants will be asked to consent to take part in the study prior to becoming pregnant. Informed consent will be obtained by the researcher or research midwife assigned to the study. The participants will be invited to contact the researcher as soon as possible after a positive pregnancy test to enable randomisation, the aim being to achieve randomisation the same day or as soon after as possible. A card with the researcher's contact details will be provided. Randomisation into one of the two study groups will be carried out by the study statistician using an independent computerised randomisation system with a randomly sized block design with block sizes of 2, 4 and 6. The study population will be stratified for those receiving concurrent treatment for RM, those with underlying medical conditions which are causative of RM, and number of previous miscarriages.

Participants will be randomised into one of the two study groups. The intervention group will be asked to use the PRCI and receive a weekly questionnaire assessment from the date of a positive pregnancy test until 12 weeks of pregnancy. The PRCI will be sent to the participant at randomisation. The control group will receive a weekly questionnaire assessment from the date of a positive pregnancy test until 12 weeks of pregnancy.

If a participant experiences a further miscarriage during the study period, they will be asked to notify the researcher. Questionnaire data from women who experience another miscarriage before 12 weeks of pregnancy analysis will be included in the data analysis.

Questionnaires will be returned by post to the research team monthly in prepaid envelopes.

For the purposes of this study, the waiting time period has been defined as the period from a positive pregnancy test until 12 weeks of pregnancy. An early pregnancy ultrasound scan is normally performed at approximately 12 weeks gestation, as part of routine antenatal care throughout the UK.

### Materials

#### Preintervention demographic questionnaire

This questionnaire will be used to obtain demographic information (eg, age, educational status), medical conditions (eg, comorbidity associated with RM), gynaecological history (eg, fertility history) and reproductive history (eg, live births and dates and number of miscarriages) in order to appraise the clinical characteristics of study participants.

The following questionnaires will be completed from randomisation, that is, on the day of a positive pregnancy test and then at weekly intervals until the woman is 12 weeks pregnant:

*Hospital Anxiety Depression Score (HADS)*:^27^ This questionnaire is commonly used by clinicians to determine the levels of anxiety and depression a patient is experiencing. The HADS consists of 14 items (7 items for each of the subscales relating to anxiety and depression) and rated on a four-point Likert scale; the total score is the sum of the 14 items, and the subscale score is the sum of the respective seven items. The scores on each subscale are interpreted in ranges 0–7 (normal), 8–10 (mild), 11–14 (moderate) and 15–21 (severe).

*The Daily Record Keeping Form (DRK)*^28^ will be used to assess emotions, appraisals, coping and physical symptoms experienced during the waiting period. The original measure was developed for daily assessments, but due to the burden of daily monitoring and potential reactivity as reported in prior research using the PRCI tool^29^ the DRK will be used only at weekly intervals in the current study. To avoid confusion for research participants it will be called the ‘Weekly Record Keeping Form’ for the duration of the study. The DRK has been shown to be sensitive to changes in emotional^13^ and physical reactions^28^ during the waiting period prior to a pregnancy test.

### Data analysis

Descriptive statistics with CIs will be used to explore the feasibility of the study procedures (numbers of eligible women, recruitment and retention rates, missing data) for each centre. Psychological well-being measures and the DRK will be summarised, and changes over the time course of the study examined descriptively. The relationship between physical symptoms, psychological well-being, appraisals and coping will also be explored descriptively using methods such as graphical displays. An assessment of any indication of an intervention effect will be made, but this will be viewed with caution given that this is a feasibility study. The variation of the psychological measurements within the control group will be used to inform power calculations for a future RCT, should this be warranted.

#### Qualitative process evaluation

The qualitative process evaluation will employ semistructured interviews using a topic guide to address relevant aspects of study objectives.

#### Proposed sample size and sampling strategy

Participants will be selected purposively, based on study group, that is, control or intervention, ongoing pregnancy or miscarriage, and clinically important demographics including age, comorbidity/medical conditions and previous live births. This method of sampling is commonly used in qualitative research^30^ and is a means of collecting perspectives from as diverse a group as possible, which is particularly important when trying to understand whether an intervention is acceptable to a population.

Sample sizes in qualitative studies vary^31^ and tend to aim for data saturation, that is, that point at which no new information is found, as the ideal. In this study, in which the aim is to study participants’ experience of study processes, rather than the development of explanatory theory, a maximum of 20 participants will be interviewed unless saturation is deemed to be achieved before this point, as it is likely a sample of this size will provide sufficient data to identify key issues and themes.

#### Data collection

Data will be collected using face-to-face, semistructured interviews and will take place at a location convenient for the participant. All the interviews will be performed by SB. Interviews will follow a topic guide which will be developed based on the study aims, a previous PRCI evaluation tool, a review of current literature, and discussion with Patient Public Involvement representatives and the supervisory team. The interview will last no longer than 60 min and will be audio-recorded and transcribed verbatim.

Participants will be eligible for interview once they reach 12 weeks of pregnancy and have completed the use of the PRCI and weekly questionnaire assessment, or in the case of the control group, weekly questionnaire assessment. If a participant experiences a further miscarriage they may still be approached for interview, but care will be taken to allow a suitable time period to elapse before this is arranged.

#### Data analysis

The aim of qualitative analysis is to unravel the plethora of data and make sense of the phenomena under investigation. Most qualitative approaches involve some degree of thematic analysis, and although thematic analysis can be as simple or complex as the research study demands, it is a more complicated process than merely coding and categorising data, and requires the researcher to ask more complex questions such as how the codes relate to each other.^32^ Therefore, in order to promote and facilitate a more systematic and robust analysis of the interview data and to aid in its presentation, the transcripts will be subjected to a thematic analysis using the framework approach.^33^ This approach was developed specifically to meet information needs and to provide outcomes or recommendations, and offers a highly visible and systematic approach to data analysis, showing very clearly how findings are derived from the data. It allows for both an inductive and exploratory approach, as other forms of qualitative analysis do, so that the integrity of individual accounts is preserved, but is also designed to facilitate analysis of specific concepts and issues that are particularly important to address, as in the proposed process evaluation.^34^

In order to further promote integrity and reliability during the data analysis process, other strategies will be introduced. Field notes will be written immediately after the interview and a reflective diary maintained, aiming to reduce the potential for the researcher’s values, beliefs and preconceptions to influence subsequent findings.^35^ Coding and analysis of the qualitative data will be completed by the researcher. In order to monitor and limit subjectivity, two members of the research supervision team will examine parts of the transcripts to compare their perceptions of the interview data and analysis with the researcher’s interpretation.

## Discussion

RM is an extremely distressing condition. Even after thorough investigation, aetiological causes can be found in only 50% of cases.^2^ Frequently, the cause is elusive or multifactorial,^36^ leading to intense frustration for the affected woman and her healthcare team, as there is currently no effective treatment or therapy which can be offered. In order to address this condition appropriately and effectively, there is a need to develop a greater understanding of the causes, treatment and effects of RM. Current research into RM focuses on aetiology and the development of medical interventions to treat the condition. Other studies have reviewed the use of interventions to alleviate distress in the period immediately following miscarriage^5^ ^37^ but there is insufficient evidence relating to the effectiveness of support during the initial waiting period of a subsequent pregnancy^3^ or the way in which such support should be delivered. This study is the first step in a programme of research concerned with improving psychological well-being for women affected by RM during the early stages of a new pregnancy; a waiting period which previous studies have indicated is associated with high levels of anxiety and distress for the affected woman, but a time period when there is limited support and therapy available. As such, it is envisaged that this research will both enhance and complement ongoing studies within a wider programme of research into the care and management of women who experience RMs.

This study is both timely and topical, investigating an issue which causes distress and anxiety to women affected by RM, and one which is proving a challenge to the healthcare professionals caring for them. The study will add valuable information to the body of evidence relating to how women who have suffered RM experience the waiting period of a new pregnancy. Additionally, the evaluation of the acceptability and feasibility of the PRCI will explore and identify how women who have experienced RM utilise this type of self-administered tool. If the PRCI proves to be an acceptable and valuable intervention then this model of care has the potential to be made more widely available within the National Health Service, both locally and nationally, as an effective, low cost, convenient, safe and easily deliverable intervention. The primary objective of this study, which lays the foundation for possible future trials, is to complete a necessary preliminary stage in the process of testing the effectiveness of the PRCI in improving quality of life for women in the initial 12 weeks of pregnancy following repeated miscarriages. The PRCI has the potential to provide a low-cost intervention to provide much needed support to a vulnerable patient population.

### Ethical considerations

This research will involve a potentially vulnerable patient group that has experienced RM. Care will be taken from the outset to ensure the health and well-being of the research participants, and they will be reminded that their participation is entirely voluntary and that they are free to withdraw at any time without their healthcare being affected. Potential participants will be given as much time as they need to decide whether they wish to take part in the study, and appropriate informed consent procedures will be adhered to.

Previous studies have demonstrated that women using the PRCI, strongly recommend its use to others,^19^ and that women who had experienced miscarriage did not report any negative experiences from using the PRCI.^3^ As such, any negative effects regarding the safety and well-being of participants in this study due to the specific use of the PRCI are not anticipated. Qualitative interviews will be arranged at a convenient time and location for the participant and will be conducted in a sensitive manner. If the participant shows any sign of fatigue or distress, then the interview will be stopped and either concluded, or agreement sought to resume at a later date.

Although this study is investigating a sensitive subject with a potentially vulnerable group, previous experience of researching sensitive topics in reproductive health suggests that women appreciate the opportunity to contribute to an important and personally relevant field of investigation.

### Trial management and monitoring

Trial management meetings will be held every 6 weeks between the researcher and the supervisory team to discuss study progress. Additionally, a steering group consisting of the researcher, the academic supervisors and the two members of the patient and public representative team will meet six monthly to scrutinise and review progress of the research. Financial management of the study will be overseen by the Research and Development Finance Manager, University Hospital Southampton.

### Dissemination

Study findings will be disseminated to study participants and through peer reviewed journals/publications, and national and international conference presentations. The Miscarriage Association has agreed to disseminate and publish study findings to the public and lay groups through their website.
